# Brain Network Controllability and Its Regional Gene Expression Profile Associated With Suicidal Tendencies in Major Depressive Disorder Patients

**DOI:** 10.1155/da/6529148

**Published:** 2025-12-05

**Authors:** Xinyi Liu, Cancan He, Dandan Fan, Sangni Liu, Haisan Zhang, Zhijun Zhang, Hongxing Zhang, Chunming Xie, Xianxian Zhang

**Affiliations:** ^1^ Department of Neurology, Affiliated ZhongDa Hospital, School of Medicine, Southeast University, Nanjing, 210009, Jiangsu, China, seu.edu.bd; ^2^ Department of Neurology, Neuropsychiatric Research Institute, School of Medicine, Southeast University, Nanjing, 210009, Jiangsu, China, seu.edu.bd; ^3^ Department of Radiology, Henan Provincial Mental Hospital, Xinxiang Medical University, Xinxiang, 453003, Henan, China, xxmu.edu.cn; ^4^ Key Laboratory of Developmental Genes and Human Disease, Southeast University, Nanjing, 210009, Jiangsu, China, seu.edu.bd; ^5^ Henan Key Laboratory of Psychology and Behavior, Xinxiang Medical University, Xinxiang, 453003, Henan, China, xxmu.edu.cn; ^6^ School of Psychology, Xinxiang Medical University, Xinxiang, 453003, Henan, China, xxmu.edu.cn; ^7^ Department of Neurology, Affiliated Yancheng Hospital, School of Medicine, Southeast University, Yancheng, 224008, Jiangsu, China, seu.edu.bd

**Keywords:** average controllability, diffusion tensor imaging (DTI), major depressive disorder (MDD), network-based statistics (NBS), weighted gene coexpression analysis (WGCNA)

## Abstract

Suicidality is the most severe sign of major depressive disorder (MDD). However, network‐wide structural dynamics alterations and transcriptional patterns in MDD patients with suicidality remain unclear. A total of 124 participants were recruited, including 31 MDD patients without suicidal ideation (MDDNSI), 34 MDD patients with suicidal ideation (MDDSI), 22 MDD patients with suicidal behavior (MDDSB), and 37 healthy controls (HCs). All participants completed diffusion tensor imaging scans and suicidal assessment. Network‐based statistics (NBSs) were used to compare differences in structural connectivity, while average network controllability (ANC) reflecting brain state dynamics was measured based on suicidality‐based circuits. Weighted gene coexpression network analysis (WGCNA) identified gene modules associated with neuroimaging phenotypes. Gene functions and cellular types in MDD patients were determined with enrichment analysis and cell‐type‐specific expression analysis. The results showed that in the suicidal ideation (SI) circuit, MDDSI patients represented decreased ANC compared to MDDNSI patients, with upregulated genes related to neuronal bundles and downregulated genes related to ribosomes. In the suicidal behavior (SB) circuit, MDDSB patients exhibited reduced ANC compared to MDDNSB patients, with upregulated genes associated with cytoplasmic pathways and positive regulation of signaling and downregulated genes related to synaptic signal transmission. Importantly, genes in both the SI circuit and SB circuit were enriched in gamma‐aminobutyric acid (GABA) neurons. The study highlights that the differential ANC of the SI network and the SB network exerts a distinctive influence on brain state trajectories in MDD patients and identifies the specific transcriptional pattern and cell types associated with brain regions related to SI or SB in depression.

## 1. Introduction

Major depressive disorder (MDD) is a common psychiatric disorder associated with severe physical and mental disability, with a global lifetime prevalence of approximately10% [1]. Previous studies have shown that 3%–8% of individuals with MDD die by suicide [2]. Suicide mainly involves suicidal ideation (SI) and suicidal behavior (SB), with prevalence risk rates of 31% and 53.1%, respectively [3, 4]. As the most severe clinical manifestation of MDD, SB exacerbates disease chronicity and predicts poorer treatment response and long‐term prognosis [5, 6]. Therefore, a deeper understanding of reliable predictors and biological factors of suicidal individuals with MDD may be essential for developing therapeutic and preventative strategies.

Recently, increasing evidence has shown that network controllability derived from the anatomical network structure can be used to clarify whether and how specific nodes constrain or facilitate dynamic changes in brain state trajectories [7, 8]. Prior empirical evidence from network control theory also proposed the formal framework that the network controllability can be efficiently characterized by how neural activity will react to an external stimulus and where the brain dynamics are constrained by a structural brain network built from the white matter (WM) tract connections [9, 10]. Network controllability represents the ease of driving the brain into desired mental states that support diverse cognitive demands based on the time‐varying structural connectome [11]. While studies suggest reduced global average network controllability (ANC) in MDD patients compared to healthy controls (HCs) [12], this metric shows no direct correlation with cross‐sectional symptom severity [13]. This apparent paradox highlights the need to investigate state‐specific network dynamics, particularly in circuits associated with critical clinical phenotypes like suicidality. Individual variation in network controllability could serve as a powerful tool for quantifying the influence of the brain’s large‐scale, dynamic network state transitions, and precisely predicting treatment response in MDD patients [14]. However, it remains unclear how network controllability dynamically influences network state transitions associated with suicidality in MDD patients.

Accumulating evidence has shown that suicidal tendencies in MDD patients are the result of complex interactions between genetic susceptibility and environmental factors [15]. In genome‐wide association studies (GWASs) or meta‐analyses, three loci that met the criteria for significance have been identified [16]. Several SB studies have identified gene associations such as glucocerebrosidase 3 (GBA3) and NLGN1 (neuroligin1) [17, 18]. However, the majority of these genome‐wide significant results for SB susceptibility studies are inconsistent or lack replication [19]. Most of these studies have focused only on the identification of differentially expressed genes (DEGs), ignoring the substantial interconnectivity between genes with comparable expression patterns in various anatomical background regions. The systems biology algorithm of weighted gene coexpression network analysis (WGCNA) has been used to assess the association between genetic architectures and WM structural networks by constructing scale‐free gene coexpression networks [20]. Previous studies have shown that regional gene expression in healthy individuals predicts loss of WM connectivity in Parkinson’s disease, Huntington’s disease, and schizophrenia [21, 22]. In addition, gene expression is theoretically closer to the neuroimaging profile of MDD than genetic variation [23]. Accordingly, describing potential changes in regional gene expression linked to connectivity loss in MDD patients with suicidal tendencies could help in illuminating the biological mechanisms that interact between networks. Furthermore, these genes in the brain can be expressed in multiple cell types and may perform different functions in each cell type [24]. Only one study has shown that the connectivity changes associated with MDD patients with SI (MDDSI)/MDD patients with SB(MDDSB) were spatially correlated with gene expression related to cellular metabolism and synaptic signal transmission [25]. However, to date, the genetic basis and cellular expression types of SI‐ and SB‐related brain WM circuits in MDD patients have not been thoroughly investigated.

The purpose of this study was to investigate how the functional alterations of network controllability that arise from structural connectivity facilitate brain dynamics and their genetic architecture in MDD patients with suicidal tendencies. We first explored whether the MDDSI or MDDSB brain is controllable and whether it can control brain state transitions in MDD patients with SI or SB. Second, the whole‐brain WM networks and subnetwork levels for controllability differences and behavioral significance were investigated according to the SI/SB circuits based on the structural networks. Third, using the Allen Human Brain Atlas (AHBA) gene expression data and enrichment analysis, we checked whether regional gene expression could account for the susceptibility to connectivity loss in specific brain regions in MDD patients. These findings identified gene expression patterns to clarify the biological and cellular processes driving the loss of connectivity associated with suicidal tendencies in depression patients.

## 2. Materials and Methods

### 2.1. Participant Recruitment and Selection Criteria

A total of 129 participants, including 90 MDD patients and 39 HCs, were enrolled in this study between August 2019 and December 2020 at the Second Affiliated Hospital of Xinxiang Medical University. The inclusion and exclusion criteria for the subjects are detailed in Supporting Information 1. After further excluding participants with overmovement and incorrectly matched echo‐planar imaging (EPI) scans, we eventually included 87 MDD patients and 37 HC subjects in our analyses. The subject protocol was approved by the Ethics Committee of the Second Affiliated Hospital of Xinxiang Medical University (Number 2017–08), and each participant voluntarily signed an informed consent form before inclusion in the study.

MDD patients were divided into three groups: Those with SB (MDDSB, *N* = 22, defined as self‐destructive acts committed with some degree of intent to end one’s life within the past year), those with suicidal ideation (MDDSI, *N* = 34, defined as thoughts or a desire to die by suicide, as assessed by Columbia‐Suicide Severity Rating Scale (C‐SSRS; ideation score ≥ 3) and persisting beyond a single transient occurrence within the past 2 weeks), and non‐SI (MDD patients without suicidal ideation [MDDNSI], *N* = 31, defined as the absence of any lifetime history of SI or SB, confirmed by C‐SSRS). The classification was determined by two independent trained psychiatrists using a structured clinical interview incorporating the C‐SSRS to ensure standardized and objective assessment. Discrepancies were resolved by consensus. The inclusion criteria for each subgroup were further verified based on the patient’s medical history, medical records, and physical examinations.

### 2.2. Behavior Assessments

The severity of depressive and anxious symptoms was assessed using the 17‐item Hamilton Depression Scale (HAMD‐17) and the Hamilton Anxiety Scale (HAMA), respectively. SI and SB were specifically assessed using the C‐SSRS to quantify severity and define groups objectively. The severity of childhood maltreatment was assessed with the Child Trauma Questionnaire (CTQ) [26], while self‐esteem was measured using the Rosenberg Self‐Esteem Scale (RSES) [27]. Cognition was assessed by the Montreal Cognitive Assessment (MoCA) [28]. The emotion control process was assessed using the Emotion Regulation Questionnaire (ERQ) [29]. The regulatory focus direction of each individual was evaluated by the Regulatory Focus Questionnaire (RFQ) [30].

### 2.3. Structural Connectivity Data

#### 2.3.1. Imaging Data Acquisition

All neuroimaging data were acquired on a 3.0 T scanner (Siemens, Erlangen, Germany), with a standard 12‐channel head coil. Diffusion tensor imaging (DTI) was obtained using spin‐echoed EPI (SE‐EPI): repetition time (TR) = 10,000 ms; echo time (TE) = 90 ms; field of view (FOV) = 256 × 256 mm^2^; matrix = 128 × 128 pixels; maximum *b* value = 1000 s/mm^2^ in 60 noncollinear directions; and voxel size = 1 × 1 × 1 mm^3^.

#### 2.3.2. Imaging Data Preprocessing and Network Construction

The DTI raw data were first evaluated by visual inspection. The DTI images were preprocessed using the FMRIB Software Library (FSL) [31], with any distortions caused by eddy‐current effects of the diffusion gradients and head motion corrected by applying affine alignment of each diffusion‐weighted image to the *b* = 0 images. After the removal of nonbrain tissues using the BET tool, the eigenvalues and eigenvectors of the estimated diffusion tensor were calculated as the fractional anisotropy (FA) values. The graphical user interface (GUI) of the LONI quality control system allows visual inspection of DTI FA images (http://pipeline.loni.usc.edu). Then, individual brains were divided into 360 cortical regions (180 regions from each hemisphere) using the Glasser Atlas in FreeSurfer [32]. This segmentation technique benefits from accurate alignment based on a large number of participants (210 healthy individuals) and outperforms other segmentation techniques [33]. The FA images generated in the diffusion space were transformed to T1 templates in the Montreal Neurological Institute (MNI) space using a nonlinear transformation. The Glasser atlas was warped from the MNI space to the diffusion space by nearest neighbor interpolation using an inverse transform. Next, whole‐brain fiber tracking was performed in the native diffusion space of each participant using the Fiber Assignment of the Continuous Tracking (FACT) algorithm embedded in the Diffusion Toolkit (http://trackvis.org/dtk/). Fiber tracking stopped at voxels with an FA < 0.2, and the angle between the two eigenvectors of the two contiguous voxels connected by tracking was greater than 45°. FA‐weighted connections were stored as 360 × 360 matrices. DTI tractography files are inspected using the LONI Viewer with an online 3D visualization module (http://pipeline.loni.usc.edu).

### 2.4. Network Controllability Calculations

The control theory concept was applied to assess which regions exert the most significant influence on driving changes in brain state trajectories in different subtypes of patients. Brain network controllability refers to the ability of the brain system to transition from any initial state to any desired state in finite time via external control inputs [34]. A state is defined as the magnitude of neurophysiological activity across brain regions at a single time point [35]. Given the established controllability of the brain structure, we assume that the system follows the discrete, noise‐free linear time‐invariant model as follows:

(1) xt+1=Axt+Bkukt,

where *x* is the temporal activity of 360 brain regions and *A* (360 × 360) is the symmetric weighted streamlined structural connectivity matrix. The input matrix *B*_*k*_ (N × m) identifies control points *k* in the brain, where *k* = (*k*1,…, *k*p) and *B*_*k*_ = (*e*_*k*1_, …, *e*_*k*p_). *e*_*i*_ is the *i*th canonical vector of dimension *N*, and *p* is the number of targeted nodes. The input *u*_*k*_ (*p* × 1) is the external input.

Subsequently, the average controllability was quantified. The average controllability is the average input energy from a set of control nodes and the overall possible target state [35]. According to this definition, the average input energy is proportional to the trace ($W_{k}^{-1}$) of the Gramian inverse matrix of controllability. The Gramian of controllability is defined as follows:

(2) Wk=∑T=0∞ATBkBkTAT.

*W*_*k*_ is adopted as the measure of average controllability. Thus, the average controllability (*a*_*c*_) can be mathematically described as follows:

(3) ac=TraceWk.

### 2.5. Gene Expression Data

The AHBA data set was selected to acquire gene expression microarray data from six postmortem adult brains [36]. The atlas covers 20,737 genes, detected by 58,692 probes extracted from 3702 spatially distinct tissue samples. Since the data set contains data from only two right‐hemisphere donors, this study chose to extract samples from the left hemisphere only. We correlated expression measurements with the neuroimaging data [37]. In brief, expressed genes were indexed by annotating gene probes, filtering probes, and selecting probes to align the location of the tissue samples from the AHBA to the MNI stereotactic space of 180 brain regions in the Glasser atlas (distance threshold set to 2 mm). Subsequent normalization was performed by scaled, outlier sigmoid normalization for individual differences. Finally, the inconsistently expressed genes were filtered. A gene expression matrix (15,745 genes × 180 brain regions) was finally obtained.

### 2.6. Statistical Analysis

#### 2.6.1. Comparison of Demographic and Behavioral Variables

The Shapiro‒Wilk test was used to test for the normal distributions of each group. Differences among groups were compared by one‐way ANOVA (for normally distributed variables) or the Kruskal‒Wallis test (for nonnormally distributed variables). The chi‐squared test was used to compare categorical variables. Pearson’s correlation analysis was performed to determine the correlations between parameters. The data are shown as the mean ± standard deviation. *p* < 0.05 was set as the threshold for statistical significance.

#### 2.6.2. Network‐Based Statistic (NBS) Analysis

To assess whole‐brain edgewise differences between each pair of groups, we performed NBS analysis on the structural brain networks [38]. In the SI circuit, those with or without SI were compared. In the SB circuit, those with or without SB (MDDNSB, merging those with and without SI into a subgroup) were compared. In brief, we conducted a two‐sample *t*‐test at each edge for significant differences between pairwise groups and then applied a primary component‐forming threshold (*p* < 0.001, uncorrected) to generate a set of suprathreshold connections. We then identified any connected components or subnetworks in the super‐threshold edge set. The statistical significance of each observed component size was assessed based on the empirical null distribution of the maximum component size obtained under the null hypothesis of random group membership (5000 permutations). A familywise error rate (FWER)‐corrected *p*‐value of <0.05 for each subnetwork was considered significant. Finally, the significant subnetworks were visualized by BrainNet Viewer (www.nitrc.org/projects/bnv/). To examine the effects related to behavioral indicators, we conducted partial correlation analysis with age, sex, education, and medication load index as nuisance covariates (*p* < 0.05, Bonferroni correction).

#### 2.6.3. Network Controllability Comparison

Statistical comparisons of ANC across different groups, as well as within the SI and SB circuit frameworks, were conducted using ANOVA if the data followed a normal distribution or the Kruskal‒Wallis test if the data deviated from normality. Post hoc analyses between groups were performed with false discovery rate (FDR) correction to identify the sources of differences. Subsequently, Pearson correlation analysis was employed to investigate the relationships between FA in brain regions exhibiting differences and behavioral scale scores and to clarify how medication types and dosages might influence ANC. Additionally, to directly clarify how medication types and dosages influence ANC, we analyzed their effects while deliberately excluding the medication load index as a covariate.

#### 2.6.4. Weighted Gene Coexpression Network Construction and Identification of Modules

Gene coexpression was analyzed using the WGCNA package in R4.0.3 to find clusters (modules) enriched with expression‐related genes [20]. First, coexpression relationships in all data sets were assessed using Pearson correlation analysis. Based on the topological overlapping dissimilarity of the network connection strengths, the adjacency matrix was transformed into a topological overlapping matrix (TOM). Next, average‐linkage hierarchical clustering was performed for each TOM to determine the individual modules. To obtain the correct module number and elucidate gene interactions, the soft threshold power used for network construction was determined by scale‐free topological analysis (*R* ^2^ > 0.85). The minimum module size was set to 100 genes, balancing biological interpretability with computational efficiency, consistent with established practices in brain transcriptomic studies [20]. Module–trait associations were assessed using Pearson correlations with Benjamini–Hochberg FDR correction (*α* = 0.05) for multiple testing across all module–trait combinations. Once the modules of interest were selected, each significant gene significance (GS) and module membership (MM) could be determined. A correlation threshold of > 0.8 for gene MM (which maximizes mean network node connectivity) and > 0.5 for gene GS was used to screen for key genes in each module. Additionally, we conducted the analysis by excluding the medication load index as a covariate.

#### 2.6.5. Gene Enrichment Analysis of the Hub Module

Pathway enrichment analysis of the genes involved in the hub module was conducted using Metascape (http://metascape.org/) to determine the biological significance of the modules. Terms with a *p*‐value <0.01, a minimum overlap of 3, and a minimum enrichment factor > 1.0 were collected. The top 5 terms were selected for visualization.

#### 2.6.6. Cell‐Specific Expression Analysis of the Hub Module

Expression‐weighted cell‐type enrichment (EWCE) analysis was performed using the R package to determine whether genes from the MDD patient subtype network modules could map to specific cell types. EWCE calculates the specificity of the target list (consisting of genes from the SI‐ and SB‐related modules) and then calculates method‐adjusted specificity *p*‐values from multiple tests via bootstrap lists. Transcriptional data were obtained from AIBS (https://portal.brain-map.org/atlases-and-data/rnaseq), including data from the middle temporal lobe [39].

#### 2.6.7. DEG Analysis and Validation of Candidate Overlapping Genes

To screen more effective genes, we used a high‐throughput Gene Expression Omnibus (GEO) data set to obtain RNA‐seq data from SI/SB circuit‐related patients, performed DEG analysis, and identified genes with overlapping correlation differences as candidate genes. There were two data sets, GPL2832 and GPL15520, from the HCs, MDDNSB patients, and MDDSB patients, and no data sets related to the MDDNSI or MDDSI patients (see Supporting Information 1: Table S3).

ROC analysis was conducted to assess the diagnostic performance parameters of the differentially overlapping genes. All the statistical analyses were considered significant when *p* < 0.05.

## 3. Results

### 3.1. Demographic and Clinical Characteristics

As shown in Table 1, Supporting Information 1: Tables S1 and S2, there were no significant differences in age, sex, or education level among the four groups. Compared with HCs, patients in the MDD group exhibited significantly elevated HAMD, HAMA, RSES, ERQ‐CR, and RFQ‐promote scores. Notably, the MDDSI group showed higher HAMD scores compared to the MDDSB group, which contrasts with the common clinical expectation that patients with SBs often present with more severe depressive symptoms. HAMA scores differed significantly between MDDSB and MDDSI patients and between MDDNSI and MDDSI, while MoCA scores showed no significant difference among groups (*p* = 0.2).

**Table 1:** Demographic and clinical characteristics across the entire subjects.

Variables	HC (*N* = 37)	MDDNSI (*N* = 31)	MDDSI (*N* = 34)	MDDSB (*N* = 22)	*p*‐Value
Age (years)	34.16 ± 8.74	31.07 ± 12.07	30.26 ± 11.67	30.09 ± 13.07	0.42
Gender (*F*/*M*)	22/15	14/17	21/13	8/14	0.13 ^∗^
Education	11.32±3.55	10.93±2.72	11.03±3.71	10.73±3.33	0.92
HAMD‐17	1.00 (0–2.50)	19.00 (13.50–22.50)^a^	21.00 (16.50–26.00)^b^	14.50 (10.00–18.00)^c^	<0.01
CTQ	33.00 (29.50–39.50)	38.00 (32.00–47.00)	43.00 (32.75–53.25)^b^	36.00 (30.00–50.25)	0.02
RSES	37.00 (30.00–46.00)	62.00 (50.00–67.00)^a^	62.00 (52.00–73.25)^b^	54.50 (49.00–65.25)^c^	<0.01
HAMA	1.00 (0–2.00)	15.00 (11.00–23.25)^a^	15.00 (12.00–19.75)^b^	14.50 (10.00–18.00)^c,d,e^	<0.01
MoCA	26.00 (24.00–28.00)	25.00 (21.00–26.00)	26.00 (52.00–73.25)	25.00 (22.00–26.25)	0.2
ERQ‐CR	36.00 (30.00–37.00)	26.00 (24.00–36.00)^a^	29.00 (20.50–34.25)^b^	29.00 (18.25–34.00)^c^	<0.01
ERQ‐ES	15.00 (10.00–20.00)	18.00 (11.25–22.75)	16.50 (11.50–20.25)	14.50 (9.00–22.50)	0.43
RFQ‐promote	20.39 ± 3.85	17.19 ± 3.60^a^	17.00 ± 3.52^b^	18.81 ± 3.87	<0.01
RFQ‐prevent	20.41 (18.50–23.50)	18.79 (16.00–21.00)	18.32 (15.00–19.00)	18.45 (16.75–21.00)	0.35

*Note:* Continuous variables were presented as the mean ± standard deviation (normal distribution) or median (lower quartile, upper quartile) (non‐normal distribution), and were compared using one‐way ANOVA or the Kruskal‐Wallis test, respectively. Categorical variables (e.g., gender) were compared using the chi‐square test. Post hoc multiple comparisons (demoted a–e) indicate: ^a^MDDNSI vs. HC; ^b^MDDSI vs. HC; ^c^MDDSB vs. HC; ^d^MDDSI vs. MDDSB; ^e^MDDNSI vs. MDDSB.  ^∗^ indicates a statistically significant difference (*p* < 0.05).

Abbreviations: CTQ, Childhood Trauma Questionnaire; ERQ–CR, Emotion Regulation Questionnaire–cognitive reappraisal; ERQ–ES, Emotion Regulation Questionnaire–expression suppression; HAMA, Hamilton Anxiety Scale; HAMD‐17, Hamilton Depression Scale–17 items; HC, healthy control; MDDNSI, major depressive disorder with nonsuicidal ideation; MDDSB, major depressive disorder with behavior; MDDSI, major depressive disorder with suicidal ideation; M/F, male/female; MoCA, Montreal Cognitive Assessment; RFQ, Regulatory Focus Questionnaire; RSES, Rosenberg Self‐Esteem Scale.

### 3.2. Differential Network Connectivity in SI and SB Circuits

The NBS analysis (Figure 1 and Supporting Information 1: Figure S1) revealed that the differential structural connections of the SI circuit were mainly concentrated in the ventral lateral prefrontal cortex, auditory areas, and temporal cortex. In the MDDSI group, the mean connectivity of the SI network was lower than that in the MDDNSI group, which may be related to cognitive reappraisal in emotion regulation (*r* = 0.33, *p* = 0.03). The differential structural connections of the SB circuit were more widely distributed, mainly in the dorsolateral prefrontal cortex, inferior parietal lobe, motor cortex, and visual cortex. The mean connectivity of the SB network in the MDDSB group was lower than that in the MDDNSB group, which may be associated with experiences of childhood abuse (*r* = −0.37, *p* = 0.01). The intergroup comparisons for the other NBS measures are illustrated in Supporting Information 1: Figure S2.

Figure 1Differential connections in MDD patients with suicidal tendencies. (A) Differential structural connectivity network in the SI circuits between the MDDSI and MDDNSI patients. (B) Differential structural connectivity network in the SB circuits between the MDDSB and MDDNSB patients. The nodal size corresponds to the number of associations (edges), and the edge width (gray lines) represents the edge weight. The histograms compare the average differential connection strength of the subnetworks in the SI‐ and SB‐related circuits. In each graph, the vertical bar represents the average connection strength for each group, and the error bar represents the standard error of the mean. Radar diagrams depict the behavioral significance of the average differential connection strengths of the subnetworks. The *r* ^2^ values represent the correlation power after removing the effects of group, age, sex, and education. The larger the *r* ^2^ value is, the closer to the margin edge. Red represents positive correlations, while blue represents negative correlations. Statistical note: NBS analysis was performed with a primary threshold of *p* < 0.001 (uncorrected), and family‐wise error rate (FWER) correction was applied to identify significant subnetworks (*p*FWER < 0.05).  ^∗^, denotes a statistically significant difference between two groups in the bar graphs and a significant correlation with the scale in the radar charts, respectively.

**Figure:**
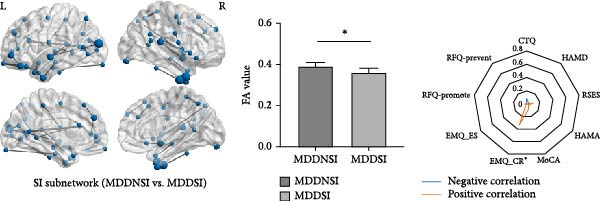
(A)

**Figure:**
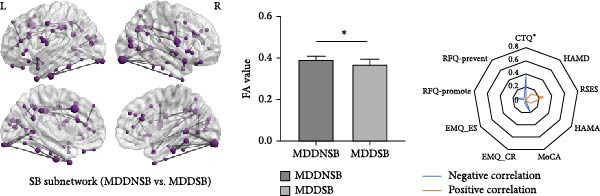
(B)

Our analysis demonstrated that the overall architecture of differential controllability networks remained regardless of whether the medication load covariate was included. However, after removing this covariate, differential controllability increased in the left temporal and right frontal lobes within the SI subnetwork and in the left frontal and bilateral occipital lobes within the SB network (Supporting Information 1: Figure S4).

### 3.3. Differential Controllability in the Framework of the SI and SB Circuits

As shown in Figure 2, the ANC differences in the four groups were mainly located in the sensorimotor areas, anterior cingulate gyrus, and frontal middle gyrus. Within the SI circuit framework, the brain regions with differences in average controllability were mainly located in the auditory association area, superior parietal lobe, lateral temporal lobe, and temporo‐parieto‐occipital area, with an increase in the ANC in the MDDNSI group and a decrease in the ANC in the MDDSI group compared to the HC group. These differences were mainly related to the severity of depression (*r* = 0.09, *p* = 0.05). Within the SB circuit framework, greater ANC differences in the MDDNSB group compared to the HCs were mainly located in the primary visual cortex, inferior parietal lobe, superior parietal lobe, middle temporal complex, and inferior frontal lobe. However, the controllability of the MDDSB group was reduced compared to that of the HCs. These differences were associated with depression severity (*r* = 0.45, *p* < 0.01), anxiety severity (*r* = 0.31, *p* < 0.01), and rumination (*r* = 0.28, *p* = 0.01). The *F* values of the ANC corresponding to the different brain regions are detailed in Supporting Information 2: S1.xlsx.

Figure 2Differential global and regional average network controllability (ANC) patterns and behavioral significance in MDD patients with suicidal tendencies. (A) Brainwide ANC differences and behavioral significance in HCs and MDDNSI, MDDSI, and MDDSB patients. (B) Differences in the ANC within the suicidal ideation framework in the HC, MDDNSI, and MDDSI groups. (C) Differences in the ANC within the suicidal behavior framework in the HC, MDDNSB, and MDDSB groups. Violin plots showing the differences in the ANC for each group within each frame. Dashed lines represent the median, quartiles, and data distribution. Radar plots depict the behavioral significance of the ANC for all participants within each frame. Statistical note: Group differences in ANC were assessed using ANOVA or Kruskal–Wallis test, followed by post‐hoc FDR correction. Correlation analyses were Bonferroni‐corrected (*p* < 0.05).  ^∗^, denotes a statistically significant difference between two groups in the violin plot and a significant correlation with the scale in the radar charts, respectively.

**Figure:**
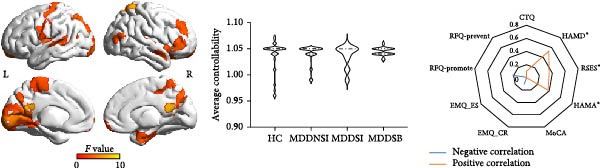
(A)

**Figure:**
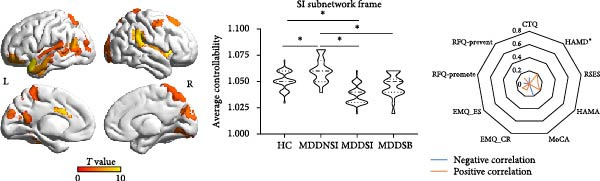
(B)

**Figure:**
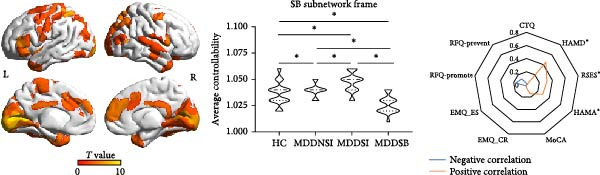
(C)

### 3.4. Identification of Modules in the Weighted Coexpression Network

We used WGCNA package clustering for the coexpression modules. Based on the results of the network topology analysis, a soft threshold of 12 (Supporting Information 1: Figure S3) was selected for subsequent network construction and module detection, and 10027 genes were clustered into eight coexpression modules labeled with different colors (Figure 3A). The hierarchical clustering of the module‐centered genes summarizes the modules generated in the cluster analysis, which combined similar modules (Figure 3B). The details of these modules are as follows: the turquoise module contains 780 genes, the black module contains 1064 genes, the blue module contains 967 genes, the red module contains 2021 genes, the yellow module contains 977 genes, the brown module contains 744 genes, the green module contains 1137 genes, and the gray module contains 940 genes. The TOM heatmap (Figure 3C) represents the interactions between the genes of the eight modules. In the SI circuit, the blue module was positively correlated with SI (*r* = 0.51), while the gray module was negatively correlated with SI (*r* = 0.36). We identified 329 key genes in the blue module and eight key genes in the gray module. In the SB loop, the red module was positively correlated with SB (*r* = 0.24), while the brown module was negatively correlated with SB (*r* = 0.18). We identified 21 key genes in the red module and 237 key genes in the brown module (Figure 3D, Supporting Information 3: S2.xlsx). The SI subnetwork exhibited robustness, as the blue and gray modules were identified both with and without covariate regression; the blue module conserved 84% of its genes, while the gray module showed little overlap (Supporting Information 1: Figure S5, Supporting Information 4: S3.xlsx). In stark contrast, the SB subnetwork displayed completely different modular architectures under the two conditions.

Figure 3Identification of modules associated with the clinical traits of MDD patients with suicidal tendencies. (A) Clustering dendrogram of the gene modules. In the WGCNA, 15,928 unique gene features were clustered into eight coexpression modules using a gene‒gene correlation model. (B) Heatmap of the correlations between module eigengenes. (C) Heatmap of the correlation between module eigengenes and clinical traits presented in the SI/SB subnetwork. Each module is annotated with the correlation value and *p* value and colored according to the correlation. (D) Module membership vs. gene significance in the “blue,” “gray,” “red,” and “brown” modules. (E) Gene–gene interaction network and description in the SI/SB subnetworks. (F) Expression‐weighted cell type enrichment (EWCE) for the SI/SB subnetwork‐associated modules. The data are presented with standard deviations from the mean.  ^∗^Statistically significant results with FDR correction. Note: Module–trait correlations were assessed using Pearson correlation with Benjamini–Hochberg FDR correction (*p* < 0.05). Gene significance (GS) > 0.5 and module membership (MM) > 0.8 were used to identify hub genes.

**Figure:**
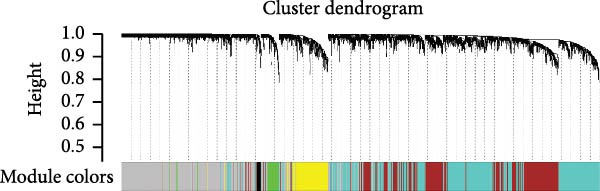
(A)

**Figure:**
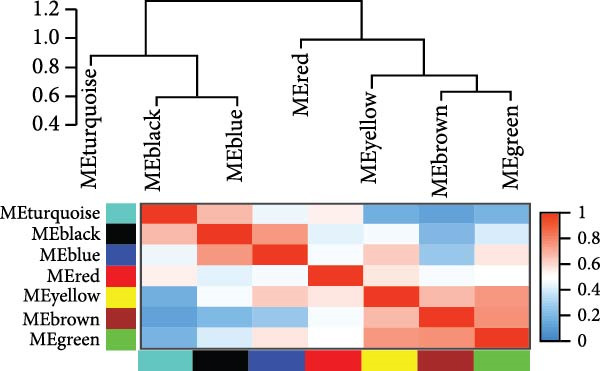
(B)

**Figure:**
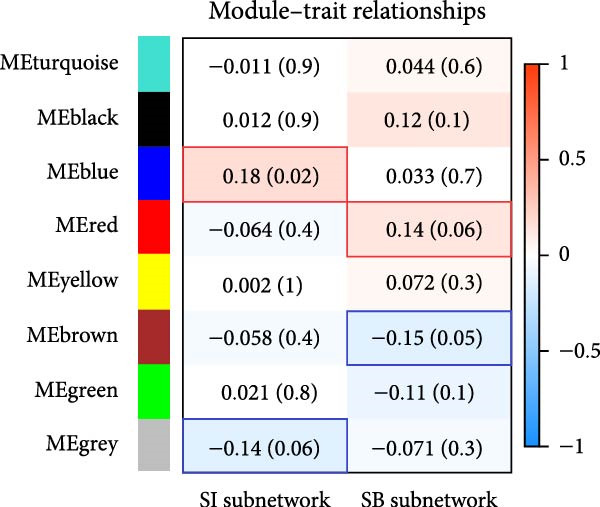
(C)

**Figure:**
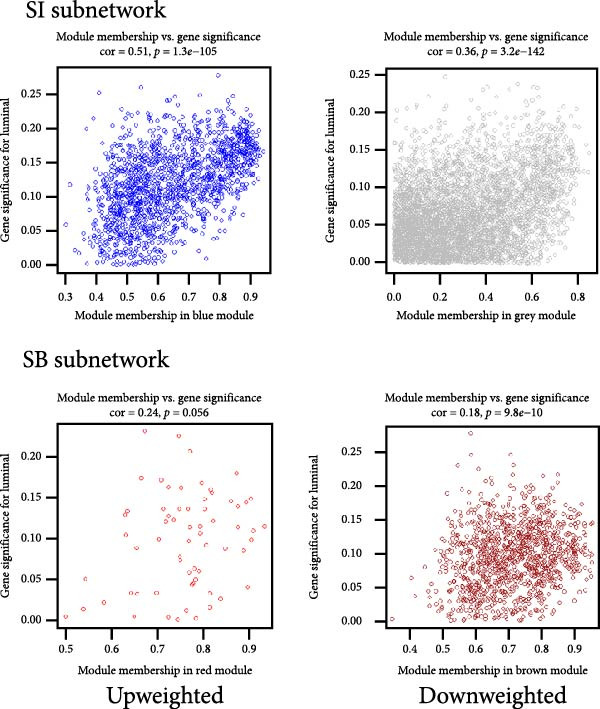
(D)

**Figure:**
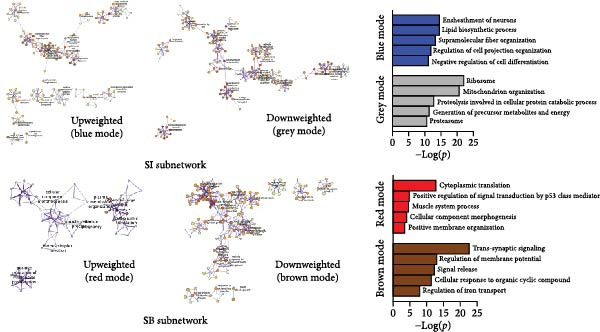
(E)

**Figure:**
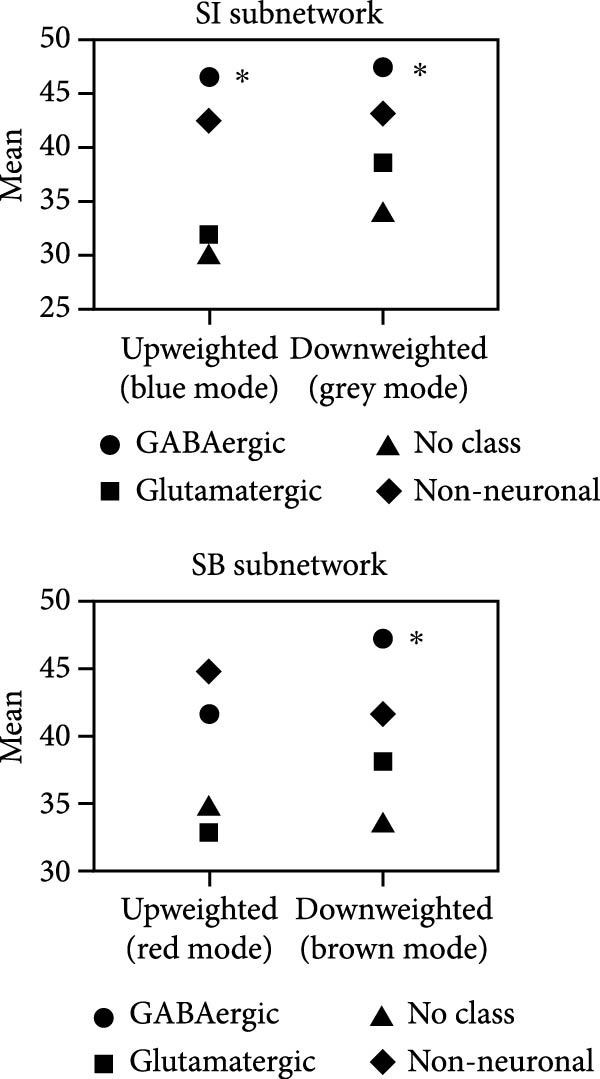
(F)

### 3.5. Functional Enrichment of Genes in the Significant Modules

For the SI (up‐weighted)‐related blue module, the enriched terms included ensheathment of neurons, lipid biosynthetic process, supramolecular fiber organization, regulation of cell projection organization, and negative regulation of cell projection organization. For the SI (down‐weighted)‐related gray module, the enriched terms included ribosome, mitochondrion organization, proteolysis involved in cellular protein catabolic process, and generation of precursor metabolites and energy. For the SB (up‐weighted)‐related red module, the enriched terms included cytoplasmic translation, positive regulation of signal transduction by p53 class mediators, muscle system processes, cellular component morphogenesis, and positive membrane organization. For the brown module associated with SB (down‐weighted), the enriched terms included transsynaptic signaling, regulation of membrane potential, signal release, cellular responses to organic cyclic compounds, and regulation of iron transport (Figure 3E).

### 3.6. Cell‐Specific Enrichment Analysis

We investigated whether genes in the SI‐ and SB‐related modules were enriched in specific cell types (Figure 3F). In the SI circuit, for both the blue and gray modules, we found that the associated genes were enriched in gamma‐aminobutyric acid (GABA) neurons. In the SB circuit, for the brown module, the associated genes were also enriched in GABA neurons; for the red module, the associated genes were not significantly enriched.

### 3.7. Validation of DEGs

In the SB circuit, we identified 163 key genes with differential expression. For the GPL2832 data set, we did not find overlapping genes. With respect to the GPL15520 data set, we found three overlapping genes between this analysis and the WGCNA, including TBX2, CLDN5, and CLEC4M. These genes differed mainly between the HC and MDDNSB groups and between the MDDNSB and MDDSB groups, with no significant differences between the HC and MDDSB groups. The spatiotemporal expression trajectories of these three genes were relatively stable across brain regions. ROC analysis revealed that TBX2 had good diagnostic performance in distinguishing between the HC and MDDNSB groups and between the MDDNSB and MDDSB groups (AUC values of 0.78 and 0.79, respectively) (Figure 4).

Figure 4RNA‐Seq verification of differentially expressed genes. (A) TBX2, CLDN5, and CLEC4M were selected for RNA‐Seq verification in an independent replication cohort of 22 patients and 65 controls. (B) Spatial‐temporal specific expression curves of three genes. (C) ROC curve of three genes between differential groups. Statistical note: Differentially expressed genes were identified with *p* < 0.05.  ^∗^, indicates a statistically significant difference (*p* < 0.05). ROC analysis was performed to evaluate diagnostic performance, with AUC values reported.

**Figure:**
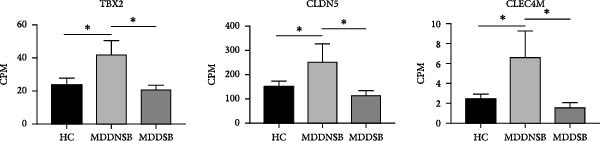
(A)

**Figure:**
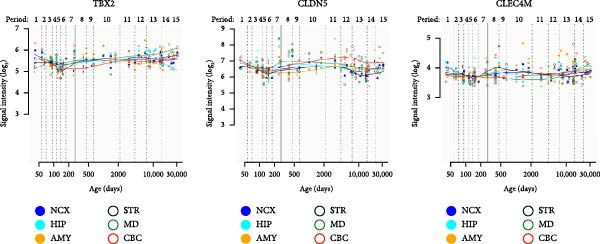
(B)

**Figure:**
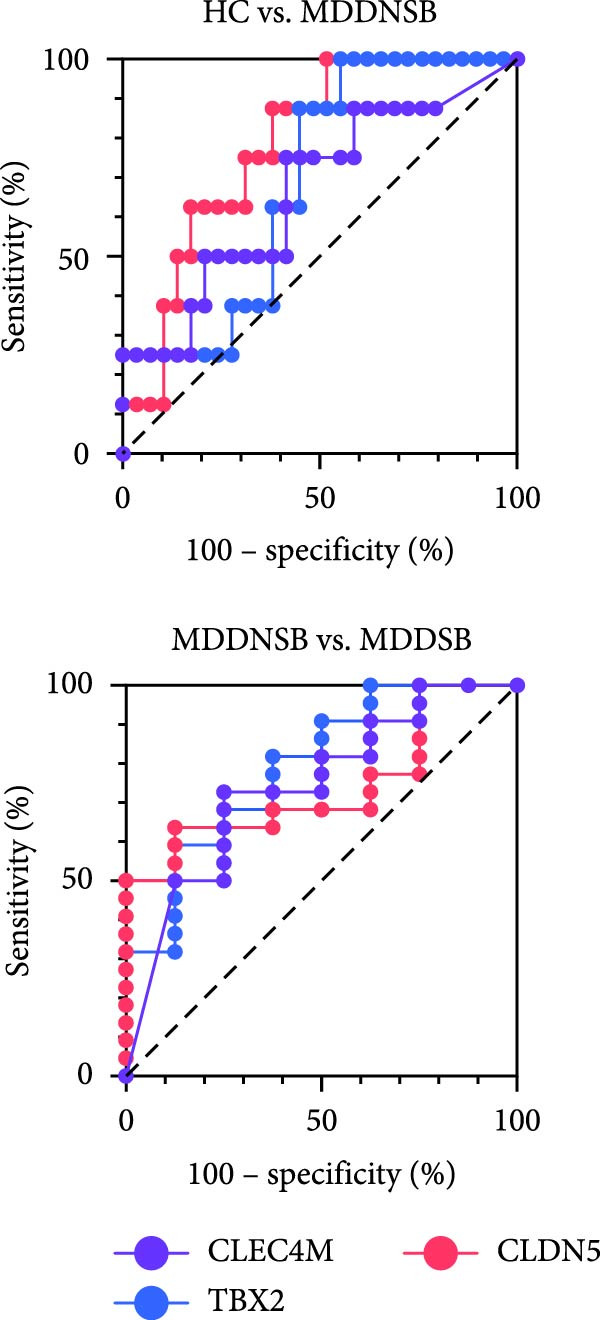
(C)

## 4. Discussion

This study is the first to explore the mechanisms of brain network transformation and gene expression patterns through SI‐ and SB‐based circuits in MDD patients. We found that in the SI circuit, network controllability was widely reduced in MDDSI patients, with up‐weighted genes related to the ensheathment of neurons and lipid biosynthetic processes, and down‐weighted genes related to ribosome and mitochondrion organization, which were enriched in GABA neurons. In the SB circuit, network controllability was reduced in both MDDSI and MDDSB patients, with up‐weighted genes related to cytoplasmic translation and positive regulation of signal transduction by p53 class mediators, and down‐weighted genes related to trans‐synaptic signaling, while up‐weighted genes were enriched in GABA neurons. Finally, TBX2 from the SB circuit has high diagnostic efficacy for MDD patients.

Within the framework of the SI circuit, the average controllability of certain brain regions, such as the auditory association area, superior parietal lobe, lateral temporal lobe, and temporo‐parieto‐occipital region, exhibits the greatest variance in driving dynamic changes in brain state trajectories. Previous studies have shown that auditory processing dysfunction, increased metabolism in the right superior parietal lobe, and resting‐state activation in the temporal lobe are closely linked to SI [17, 40, 41, 42, 43], and these abnormalities may impede the extraction of emotional meaning from rewarding stimuli or generate negative self‐awareness, thereby fostering negative attentional bias [44]. Hypermetabolism in the SPL could reflect aberrantly heightened attentional control [45], potentially leading to excessive self‐referential processing mediated by the DMN, thereby facilitating excessive negative self‐referential thought [46]. Future studies should explicitly test whether DMN connectivity mediates the relationship between parietal hyperactivity and attentional bias in depression. Our study revealed that the average controllability was lower in the MDDSI group than in the HC group, suggesting that MDDSI patients may have a reduced ability to modulate their brain transitions to meet various cognitive or emotional conditions. Conversely, the MDDNSI group exhibited increased average controllability. One possible interpretation is that MDDNSI patients might engage in adaptive neuroplastic changes, potentially by reallocating cognitive resources to and increasing the reliance on well‐functioning brain regions to maintain behavioral control [12], which needs to be validated by future longitudinal studies. Within the framework of the SB circuit, the average controllability was lower in the primary visual cortex, inferior parietal lobule, and superior parietal lobule in the MDDSB group than in the HC group. Suicide attempters have been found to exhibit increased cortical volume or thickness in the lateral occipital cortex, which is involved in visual information processing [47, 48]. Moreover, suicide attempters with MDD are more likely to produce negative responses when receiving emotional visual stimuli [49]. In addition, the parietal lobe is the pivotal region of the attentional network that interrupts and resets salient stimuli, as well as organizes actions, makes decisions, and predicts rewards when the outcome of a choice is uncertain [50, 51]. As such, parietal dysfunction in suicide attempters may lead to impaired distraction, which may predispose suicide attempters to persistent rumination and associated emotional states, leading them to attempt to end their lives based on a narrow view of current life difficulties, coping options, and future possibilities [52].

After conducting a gene coexpression analysis focused on the SI circuit, we found up‐weighted genes associated with the ensheathment of neurons, lipid biosynthetic processes, and supramolecular fiber organization. Conversely, we observed down‐weighted genes linked to ribosomes and mitochondrial organization. Neurons are essential for the proper functioning of the nervous system, while oligodendrocytes can form myelin sheaths that wrap around axons to aid neurotransmission and regulate neurometabolic homeostasis [53]. Disruption of the neurological support and regulation of myelin may lead to altered brain connectivity, reduced glutamatergic neurotransmission, and dysregulated expression of endogenous antidepressant factors, resulting in MDD [54]. Some studies have shown that the expression of fatty acid biosynthetic genes in MDD patients is reduced and may respond to defects in the compensatory mechanisms that upregulate gene expression in response to defects in long‐chain polyunsaturated fatty acids [55].

Furthermore, understanding the transcriptomic signatures of specific neuroanatomical regions at as much cellular resolution as possible is essential for dissecting the molecular basis of brain function. Previous studies have shown that the expression levels of genes associated with GABAergic or glutamatergic synaptic transmission in postmortem astrocyte samples are significantly increased in MDDSB patients, whereas the levels of these transcripts are decreased in MDDNSB patients [56]. In our research, we found that loss of SI connectivity in MDD patients was associated with genes in GABAergic neurons, and loss of SB connectivity was associated only with high‐weighted genes in GABAergic neurons. GABAergic neurons are present in all layers of the neuraxis, accounting for 20%–40% of all neurons, and are the main neurotransmitters regulating neural inhibition in the brain. Studies have shown that GABAA neuronal inhibitory signaling is responsible for subunit coordination. Emerging evidence suggests that dysregulation of GABAA receptor subunits (*α* 1, *α* 2, *α* 5, *δ*) disrupts cortical excitatory‐inhibitory balance in mood‐related neural circuits, contributing to emotional dysregulation in MDD [57]. While general GABAergic deficits correlate with core depressive symptoms [58], suicide specifically correlates with distinct patterns: selective downregulation of GABAA *α* subunits [59], unique DNA methylation profiles in GABA receptor genes [60], and altered GABA‐glutamate corelease patterns in prefrontal‐limbic circuits [61]. Thus, alterations in GABAA neuronal inhibitory signaling may be associated with depression and suicidality [56]. In addition, mutations in transport proteins regulating the GABA receptor fraction at the synapse affect mood‐related behaviors in patients [62]. Structural connections in MDD patients with suicidal tendencies rely primarily on myelin for signaling. The transcriptional profile of GABA interneurons controls the properties of synaptic connections and synaptic communication.

An additional point warrants attention: the counterintuitive finding of higher depression severity in the MDDSIs than the MDDSBs may reflect clinical heterogeneity, where suicidal acts can be driven by acute impulsivity or stressors rather than chronic depressive severity [63]. The cross‐sectional assessment might fail to capture the dynamic symptom escalation preceding a suicide attempt. Furthermore, more intensive pharmacological interventions in the MDDSBs prior to assessment may have attenuated their HAMD scores [64]. Crucially, the MDDSBs exhibited significant reductions in ANC and distinct gene expression patterns, indicating that the neurobiology of SB involves a vulnerability partly independent of depressive symptom severity.

Our study has several limitations. First, as a cross‐sectional study, it was difficult to verify the causal relationship between SI/SB and WM alterations. Second, the results of the present study were based on WGCNA data mining without further experimental evidence. Third, the AHBA gene expression data used in this study were derived from postmortem brains of neurotypical adults. While this resource provides a valuable spatial map of regional gene expression, it may not fully capture the pathological transcriptional alterations specific to MDD patients with suicidal tendencies. The use of normative expression data limits our ability to directly link the identified gene coexpression modules to disease‐specific pathophysiology. Consequently, the associations reported between regional gene expression and network controllability or connectivity loss should be interpreted as reflecting spatial correlations with baseline transcriptional architecture rather than disease‐driven changes. Future studies utilizing postmortem brain tissue from well‐characterized MDD patients with and without suicidality are crucial to validate and extend these findings. Therefore, the potential transcriptional changes in MDD patients with suicidal tendencies may be clarified using brain tissue samples from these patients with suicidal tendencies in future research.

In summary, the temporal–parietal regions of the SI network and the frontal–parietal–occipital regions of the SB network exhibited the greatest differences in driving changes in brain‐state trajectories in MDD patients. Individuals in the SI network had greater control over mood shifts as depressive symptoms increased, whereas those in the SB network were more instrumental in guiding the brain system toward behaviors such as rumination. The different suicide subtype networks of MDD patients are represented by different gene expression patterns and cell types and these key gene and cell type characteristics can help to elucidate the pathological basis of WM circuits associated with suicide. These findings provide potential targets for future clinical treatments or basic research on suicide in patients with MDD.

## Consent

All protocols received were approved by the Ethics Committee of Henan Provincial Mental Hospital Affiliated with Xinxiang Medical University (Approval ID: 2017–08). Written informed consent was obtained from all participants prior to the study.

## Disclosure

The authors confirm that permission was obtained to use the Montreal Cognitive Assessment (MoCA) scale for the purposes of this research. Proper authorization was received in accordance with the guidelines provided by the copyright holders of the MoCA. All authors have finally approved the manuscript.

## Conflicts of Interest

The authors declare no conflicts of interest.

## Author Contributions

All authors have made substantial intellectual contributions to the manuscript in one or more of the following areas: Chunming Xie and Xianxian Zhang made significant contributions to the conceptualization of the work and funding. Zhijun Zhang provided critical review and valuable intellectual content. Xinyi Liu drafted the manuscript and performed data analysis and software visualization. Cancan He was responsible for technical guidance and methodology. Dandan Fan and Sangni Liu completed the scale assessment. Haisan Zhang conducted data verification. Hongxing Zhang interpreted the data.

## Funding

This work was funded by the Science and Technology Innovation 2030 Major Projects (Grant 2022ZD0211600), the National Natural Science Foundation of China (Grants 82071204 and 82271574 to Chunming Xie, 82201237 to Cancan He, and 82501853 to Xinyi Liu), the Key Project of Jiangsu Commission of Health (Grant ZDB2020008), and Yancheng Medical Science and Technology Development Plan Project (Grant YK2020070), and medical research projects were approved by the Yancheng Health Commission in 2023 (Grant YK2023088).

## Supporting Information

Additional supporting information can be found online in the Supporting Information section.

## Supporting information


**Supporting Information 1** Tables and Figures: contains (1) detailed methodology (inclusion/exclusion criteria and medication load computation); (2) three excel data sets (S1.xlsx: regional controllability FF‐values, S2.xlsx: suicidal tendency‐gene correlations, S3.xlsx: pretreatment hub genes); (3) three demographic/clinical tables (Tables S1–S3); and (4) five figures analyzing structural connectivity (Figures S1,S2), network controllability (Figure S4), and gene modules (FiguresS3, S5) to support the main findings. Method 1. Inclusion and exclusion criteria for MDDs and HCs. Method 2. Computation of medication load index. Xlsx S1: value of average controllability corresponding to different brain regions. Xlsx S2: Gene correlation with Suicidal tendency trait. Table S1: Demographic and clinical characteristics in the suicidal ideation (SI) subnetwork. Table S2: Demographic and clinical characteristics in the suicidal behavior (SB) subnetwork. Table S3: Demographics and gene expression differences in GSE101521 database. Figure S1: Circular connectivity maps of connectivity differences. Figure S2: White matter paired differential structural connectivity and behavioral domains outside the SI and SB subnetworks.

**Supporting Information 2** S1.xlsx: Value of average controllability corresponding to different brain regions.

**Supporting Information 3** S2.xlsx: Gene correlation with Suicidal tendency trait.

**Supporting Information 4** S3.xlsx: Pretreatment hub genes.

## Data Availability

The data that support the findings of this study are available from the corresponding author upon reasonable request.
