# Supplementary material for: Brain Network Controllability and Its Regional Gene Expression Profile Associated With Suicidal Tendencies in Major Depressive Disorder Patients
Source: Depress Anxiety. 2025 Dec 5;2025:6529148. doi: 10.1155/da/6529148 (PMC12714167; doi:10.1155/da/6529148)
Supplement: Supplementary file 1 — Supporting Information 1 Tables and Figures: contains (1) detailed methodology (inclusion/exclusion criteria and medication load computation); (2) three excel data sets (S1.xlsx: regional controllability FF‐values, S2.xlsx: suicidal tendency‐gene correlations, S3.xlsx: pretreatment hub genes); (3) three demographic/clinical tables (Tables S1–S3); and (4) five figures analyzing structural connectivity (Figures S1,S2), network controllability (Figure S4), and gene modules (FiguresS3, S5) to support the main findings. Method 1. Inclusion and exclusion criteria for MDDs and HCs. Method 2. Computation of medication load index. Xlsx S1: value of average controllability corresponding to different brain regions. Xlsx S2: Gene correlation with Suicidal tendency trait. Table S1: Demographic and clinical characteristics in the suicidal ideation (SI) subnetwork. Table S2: Demographic and clinical characteristics in the suicidal behavior (SB) subnetwork. Table S3: Demographics and gene expression differences in GSE101521 database. Figure S1: Circular connectivity maps of connectivity differences. Figure S2: White matter paired differential structural connectivity and behavioral domains outside the SI and SB subnetworks. [file DA-2025-6529148-s002.docx]

**SUPPLEMENTARY MATERIAL**

**Title: Brain network controllability and its regional gene expression profile associated with suicidal tendencies in major depressive disorder patients.**

Xinyi Liu^a,b^, Cancan He^a,b^, Dandan Fan^a,b^, Sangni Liu^a,b^, Haisan Zhang^c^, Zhijun Zhang^a,b,d^, Hongxing Zhang^e,f^, Chunming Xie^a,b,d,†^, Xianxian Zhang^g,†^

**Methods**

1. ***Inclusion and exclusion criteria for MDDs and HCs***

Inclusion criteria for MDD patients were as follows: (1) the MDD diagnosis was identified by trained interviewers according to the Structured Clinical Interview for Diagnostic and Statistical Manual of Mental Disorders, 4th Edition (DSM-IV)(AP., 2000) and 17-item Hamilton Rating Scale for Depression (HAMD-17)(Hamilton, 1960). MDD patients aged 18-60 years. Exclusion criteria were as follows: (1) patients with organic brain disorders; (2) patients with another DSM-IV axis I and/or II diseases, including bipolar disorder, schizophrenia, substance abuse, or a family history of mental illness; (3) drug and/or alcohol dependence; (4) patients with severe medical comorbidity; (5) pregnancy or lactation.

For HCs, the participant age range was 18-60 years and the total HAMD-17 score was less than 7. Exclusion criteria that applied to the MDD patients were also applicable to HCs participants. All participants in this study were Chinese nationals and right-handed persons.

1. ***Computation of medication load index***

This study included 29 patients with first-onset MDD and 61 patients with relapsed MDD. For the relapsed patients, 55 patients received medication. We have removed medication use as a covariate in the analysis. It was done as follows: First, the type and dosage of psychotropic medication are recorded at baseline. Each psychotropic medication is encoded as non-existent (0), equal to or below the low average dose (1), or above the average dose (2), with reference to the midpoint of the recommended daily dose range provided by the Physician's Desk Reference(Reynolds, 2008). The medication load composite measurement is calculated by summing up the medications for each individual to reflect the dosage and types of medications taken.

**S1.xlsx** F value of average controllability corresponding to different brain regions.

**S2.xlsx** Gene correlation with Suicidal tendency trait.

**S3.xlsx** Pre-treatment hub genes in suicidal ideation and behavior networks.

**Table**

**Table. S1 Demographic and clinical characteristics in the suicidal ideation (SI) subnetwork.**

|  | HC  (N=37) | MDDNSI  (N=31) | MDDSI  (N=34) | *P* value |
| --- | --- | --- | --- | --- |
| Age(years) | 29.16±11.76 | 27.07±12.08 | 26.26±11.67 | 0.28 |
| Gender(F/M) | 22/15 | 14/17 | 21/13 | 0.22^*^ |
| Education | 11.32$\pm$3.55 | 10.93$\pm$2.72 | 11.03$\pm$3.71 | 0.88 |
| CTQ | 34.76$\pm$7.49 | 40.72$\pm$11.08^a^ | 43.21$\pm$11.02^b^ | 0.002 |
| RSES | 37.95±10.01 | 58.90±13.06^a^ | 60.71±14.31^b^ | <0.001 |
| HAMD-17 | 1.32±1.38 | 18.34±6.89^a^ | 20.53±7.03^b^ | <0.001 |
| HAMA | 1.00±1.37 | 16.57±7.72^a^ | 15.09±6.87^b^ | <0.001 |
| MoCA | 25.81±3.06 | 24.21±4.70 | 25.50±3.29 | 0.19 |

**Note:** ^*^, *P* values were obtained using the chi-square test; other *P* values were obtained by one-way ANOVA; Unless indicated, data are presented as meam± standard deviation. Post-hoc analyses were used with least significance difference (LSD) correction (*P*<0.05): ^a^, statistical difference was detected between MDDNSI group and HC group; ^b^, statistical difference was detected between MDDSI group and HC group; **Abbreviations:** M/F: Male/Female; MDDSI, major depressive disorder with suicidal ideation; MDDNSI, major depressive disorder with non-suicidal ideation; HC, healthy control; HAMD-17, Hamilton Depression Scale-17 items; Childhood Trauma Questionnaire; RSES, Rosenberg Self-Esteem Scale; HAMA, Hamilton Anxiety Scale; MoCA, Montreal Cognitive Assessment.

**Table. S2 Demographic and clinical characteristics in the suicidal behavior (SB) subnetwork.**

|  | HC  (N=37) | MDDNSB  (N=65) | MDDSB  (N=22) | P value |
| --- | --- | --- | --- | --- |
| Age(years) | 29.16±11.76 | 27.63±11.77 | 28.18±12.84 | 0.45 |
| Gender(F/M) | 22/15 | 35/30 | 8/14 | 0.23 |
| Education | 11.32$\pm$3.55 | 10.98$\pm$3.27 | 10.73$\pm$3.33 | 0.79 |
| CTQ | 34.76$\pm$7.49 | 42.06$\pm$11.03^a^ | 40.82$\pm$13.63 | <0.001 |
| RSES | 37.95±10.01 | 58.90±13.06^a^ | 57.09±13.89^b^ | <0.001 |
| HAMD-17 | 1.32±1.38 | 19.52±7.00^a^ | 14.64±5.70^b, c^ | <0.001 |
| HAMA | 1.00±1.37 | 16.57±7.72^a^ | 9.77±7.09^b, c^ | <0.001 |
| MoCA | 25.81±3.06 | 24.21±4.70 | 24.23±3.69 | 0.26 |

**Note:** ^*^, *P* values were obtained using the chi-square test; other *P* values were obtained by one-way ANOVA; Unless indicated, data are presented as meam± standard deviation. Post-hoc analyses were used with least significance difference (LSD) correction (*P*<0.05): ^a^, statistical difference was detected between MDDNSB group and HC group; ^b^, statistical difference was detected between MDDSB group and HC group; ^c^, statistical difference was detected between MDDSB group and HC group; **Abbreviations:** M/F: Male/Female; MDDSB, major depressive disorder with suicidal behavior; MDDNSB, major depressive disorder with non-suicidal behavior; HC, healthy control; HAMD-17, Hamilton Depression Scale-17 items; Childhood Trauma Questionnaire; RSES, Rosenberg Self-Esteem Scale; HAMA, Hamilton Anxiety Scale; MoCA, Montreal Cognitive Assessment.

**Table.S3 Demographics and gene expression differences in GSE101521 database.**

|  | HC  (N=29) | MDDNSB  (N=9) | MDDSB  (N=21) | P value |
| --- | --- | --- | --- | --- |
| Age | 43.52$\pm$3.95 | 57.67$\pm5.17$ | 52.05$\pm$21.74 | 0.142 |
| Gender(F/M) | 23/6 | 6/3 | 13/8 | 0.002^*^ |
| Rin | 7.03$\pm0.26$ | 7.38$\pm$0.38 | 9.92$\pm$3.34 | 0.526 |
| PMI | 13.21$\pm$0.86 | 15.06$\pm$1.46 | 15.97$\pm$1.59 | 0.230 |
| Brain_ph | 6.49$\pm$0.06 | 6.41$\pm$0.11 | 6.54$\pm$0.14 | 0.775 |
| SERPINA3 | 124.89$\pm$84.70 | 114.27$\pm$87.75 | 33.23$\pm$9.77 | 0.626 |
| TBX2 | 24.02$\pm$3.75 | 42.27$\pm$8.20 | 21.03$\pm$2.46 | 0.016 |
| CLDN5 | 153.94$\pm$19.46 | 254.11$\pm$72.65 | 116.16$\pm$17.78 | 0.022 |
| CLEC4M | 2.50$\pm$0.43 | 6.69$\pm$2.57 | 1.61$\pm$0.46 | 0.003 |

**Note:** ^*^, *P* values were obtained using the chi-square test; other *P* values were obtained by one-way ANOVA; Unless indicated, data are presented as mean± standard deviation. Post-hoc analyses were used with least significance difference (LSD) correction (*P*<0.05): ^a^ statistical difference was detected between MDDNSB group and HC group; ^b^ statistical difference was detected between MDDSB group and HC group; **Abbreviations:** M/F: Male/Female; HC, healthy control; SERPINA3, Serpin Family A Member 3; TBX2, T-Box Transcription Factor 2; CLDN5, Claudin 5; CLDN5, C-Type Lectin Domain Family 4 Member M;

**Figure legends**

**Figure S1. Circular connectivity maps of connectivity differences. (A)** Regional differences in white matter (WM) networks in the MDD patients with suicidal ideation (SI) and **(B)** with suicidal behavior (SB) circuits. The outermost ring corresponds to the cortical allocation. The inner ring corresponds to the name of the specific brain region under each cortical allocation. The connecting lines in the center of the connectivity diagram correspond to the flow lines between the regions computed by WM fiber bundle imaging. **Abbreviations:** MT+_Complex MT+_Complex_and_Neighboring_Visual_Areas, SomaSens_Motor Somatosensory_and_Motor, ParaCentral_MidCing Paracentral_Lobular_and_Mid_Cingulate, Insula_FrontalOperc Insular_and_Frontal_Opercular, TPO Temporo-ParietoOccipital_Junction, AntCing_MedPFC, Anterior_Cingulate_and_Medial_Prefrontal, OrbPolaFrontal Orbital_and_Polar_Frontal.

**Figure S2. White matter paired differential structural connectivity and behavioral domains outside the SI and SB subnetworks.** Nodal size corresponds to the number of associations (edges), and edges width (grey lines) represents the edge weights. The histogram quantifies the structural connectivity of the various groups that were compared in pairs. In each graph, the vertical bar represents the average connection strength for each group; the error bar represents the standard error of the mean. Radargrams show the correlation between structural connectivity for both groups and each scale. R^2^ values are shown after removing group, age, gender, and education. The larger the r^2^ value, the closer to the margin edge. The red color represents positive correlations, while the blue color represents negative correlations. **Abbreviation:** MDDSI, major depressive disorder with suicidal ideation; MDDNSI, major depressive disorder with non-suicidal ideation; MDDSB, major depressive disorder with behavior; HC, healthy control; HAMD-17, Hamilton Depression Scale-17 items; CTQ, Childhood Trauma Questionnaire; RSES, Rosenberg Self-Esteem Scale; HAMA, Hamilton Anxiety Scale; MoCA, Montreal Cognitive Assessment; ERQ-CR, Emotion Regulation Questionnaire-cognitive reappraisal; ERQ-expression suppression; RFQ, Regulatory Focus Questionnaire.

**Figure S3. Determination of soft-thresholding power in WGCNA analysis.** (A) Scale-free topology fit index (R²) and (B) mean connectivity analysis for different soft-thresholding powers (*β*). The red arrow indicates the selected power *β* = 12, which achieved a scale-free topology fit (R² > 0.85) while maintaining biologically meaningful network connectivity. Dashed lines in (A) represent the threshold for scale-free topology (*R*² = 0.8).

**Figure S4. Differential global and regional average network controllability (ANC) patterns and behavioral significance in medication-naïve MDD patients with suicidal tendencies.** (A) Brainwide ANC differences and behavioral significance in HCs and medication-naïve MDDNSI, MDDSI, and MDDSB. (B) Differences in the ANC within the suicidal ideation framework in the HC, MDDNSI, and MDDSI groups. (C) Differences in the ANC within the suicidal behavior framework in the HC, MDDNSB, and MDDSB groups. Violin plots show the distribution of ANC values for each group, with dashed lines representing the median, quartiles, and data distribution. **Abbreviation:** MDDSI, major depressive disorder with suicidal ideation; MDDNSI, major depressive disorder with non-suicidal ideation; MDDSB, major depressive disorder with behavior; HC, healthy control; ANC, average network controllability. **Statistical note:** Group differences in ANC were assessed using ANOVA or Kruskal–Wallis test, followed by post-hoc FDR correction. All data are from baseline (pre-treatment) assessments.

**Figure S5. Identification of modules associated with the clinical traits of medication-naïve MDD patients with suicidal tendencies.** (A) Heatmap depicting the correlation between module eigengenes and clinical traits within the suicidal ideation/behavior (SI/SB) subnetwork. Each cell is annotated with the correlation coefficient and corresponding p-value, and colored based on the strength and direction of the correlation. (B-D) Scatter plots showing the relationship between module membership (MM) and gene significance (GS) for the (B) "blue," (C) "gray," (D) "red," and "brown" modules. Statistical note: All data were derived from baseline (pre-treatment) assessments of medication-naïve patients. Module-trait correlations were assessed using Pearson correlation with Benjamini–Hochberg FDR correction (p < 0.05). Hub genes were defined as those with gene significance (GS) > 0.5 and module membership (MM) > 0.8.*

**Reference**

AP., A. (2000). *Diagnostic and statistical manual of mental disorders: DSM-IV-TR. 4th ed.* Washington, DC: American Psychiatric Association.

Hamilton, M. (1960). A rating scale for depression. *J Neurol Neurosurg Psychiatry, 23*, 56-62. doi:10.1136/jnnp.23.1.56

Reynolds, C. R. (2008). Physician's desk reference. *Encyclopedia of special education*, 1572-1572.
